# Transcatheter and surgical treatment of tricuspid regurgitation: Predicting right ventricular decompensation and favorable responders

**DOI:** 10.3389/fcvm.2022.980639

**Published:** 2022-09-27

**Authors:** Alessandra Sala, Alessandro Beneduce, Francesco Maisano

**Affiliations:** ^1^Department of Cardiac Surgery, IRCCS San Raffaele Hospital, Vita-Salute San Raffaele University, Milan, Italy; ^2^Department of Cardiology, IRCCS San Raffaele Hospital, Vita-Salute San Raffaele University, Milan, Italy

**Keywords:** tricuspid regurgitation, transcatheter interventions, surgical treatment, patient selection, outcomes

## Abstract

Isolated tricuspid regurgitation (TR) has gained increasing recognition in recent years both in the surgical and in the cardiological community. Left untreated, isolated TR significantly worsens survival. Despite being a strong predictor of negative prognosis, interventions to correct TR are rarely performed due to increased surgical risk and late patient presentation. Recently, the ultimate focus has been on patient selection, surgical or transcatheter indication, and correct timing. Furthermore, of paramount importance is the identification of predictors of outcome following treatment, in order to discriminate between favorable and unfavorable responders and guide the decision-making process of the most adequate treatment for every patient.

## Introduction

Isolated tricuspid regurgitation (TR) has gained increasing recognition in recent years both in the surgical and in the cardiological community. Initially considered benign, isolated severe TR has been found to be a strong predictor of prognosis (1, 2). Furthermore, when left untreated, TR significantly worsens survival, with a mortality rate at 5 years of ~50% (3–5). Despite such strong evidence in the literature, management of patients with severe isolated TR remains controversial. Current guidelines (6, 7) provide specific indications for treatment of TR; while surgical correction of TR concomitantly to left-sided heart diseases has been accepted and is commonly performed, reluctancy remains regarding treatment of isolated TR. This is mainly related to the fact that even if severe, TR can be clinically well-tolerated for many years. Patients, in fact, tend to be asymptomatic, with a good quality of life, and whenever minor symptoms arise, they can be initially easily managed with an adequate medical therapy (8). However, following many years of tolerating TR, patients tend to develop organ failure difficult to manage with medical therapy, requiring a structural intervention on the valve which becomes high risk for the multimorbid status of the patient (9–11). For this reason, for years, an extremely high in-hospital mortality following surgery has been reported in the literature, together with great uncertainty regarding long-term outcomes (12–14).

Therefore, despite being a disabling condition, a very low percentage of patients affected by isolated TR (~5%) are currently receiving treatment, resulting in significant undertreatment of the disease (5, 15). This large unmet clinical need has favored the development and exponential growth of transcatheter devices for the treatment of TR. However, regardless the treatment strategy, whether surgical or transcatheter, patient selection and correct timing play the most important role in determining a favorable outcome following TR treatment (16). Recently, the ultimate focus has been trying to identify predictors of outcome following tricuspid valve (TV) treatment.

In the present article we aim at reviewing the currently available results in the literature regarding isolated TR treatment, both surgical and transcatheter, with particular attention to outcomes and predictors of a favorable vs. an unfavorable response.

## Surgical treatment

The majority of tricuspid valve operations are performed concomitantly to left-sided valve surgeries, while only a minority, ~14%, are performed in isolation (17–19). This likely occurs in response to the historically reported high in-hospital mortality rates following isolated TV surgery and poor long-term outcomes, that have remained relatively stable during the last decade. Previous studies have indeed reported an in-hospital mortality ranging from 8.8 to 37%, associated to a 30 day all-cause death rate ranging from 3.2 to 16% and a 5-year mortality rate of 55% (18, 20–22). Furthermore, these studies reported a trend toward increasing patient complexity over time, and a significant impact on outcomes of factors associated with disease duration and late clinical presentation (17, 19). Recent data has underlined how early referral for surgical correction results in excellent both short and long-term outcomes (23–27). These findings support the message that the cardiac surgery community has recently tried to deliver regarding “early referral and treatment” in TR. The surgical act of TV repair or replacement is not technically demanding in itself and the outcome is therefore almost exclusively dependent on the baseline patient's profile, and in particular, on right ventricular (RV) function (28) and the overall right heart physiological status. While American guidelines (6) tend to be more conservative, and suggest waiting for the development of signs or symptoms of right heart failure (RHF) before recommending TV repair or replacement (Class IIa), European guidelines (7) have recognized that surgery might be considered in patients prior to development of RV dysfunction and end-organ damage, even in asymptomatic patients, whenever there is evidence of ongoing right heart remodeling.

However, to date, the questions of when to perform isolated TV surgery for severe TR, when is referral considered “early” and when is late referral considered “too late” are of crucial importance.

Quite a few authors have tried to identify predictors of a favorable outcome in order to better aid in the stratification of surgical risk (Table 1).

**Table 1:** Characteristics of the surgical studies in the literature.

**Study**	**No. of patients**	**Age (years)**	**Procedure TVR**	**NYHA III/IV**	**REDO**	**RHF episodes**	**RHF signs**	**End-organ involvement**	**LVEF (%)**	**≥Moderate RV dysfunction**	**sPAP (mmHg)**	**Outcomes**
Dreyfus (28)	466	60 ± 16	57%	47%	24%	35%	57%	33% CKD, 12% liver disease	58 ± 9%	17%	40 ± 11	10% in-hospital mortality 38% 5–years all-cause death
Sala (26, 27)	172	66 [55–74]	75%	62.2%	57.6%	34.3%	21.5%	22.7% CKD	60% [55–60]	13.6%	40 [35–48]	5.8% in-hospital mortality 15% 5-years all-cause death
Weiss (31)	43	65.2 ± 13.8	41.9%	72.1%	27.9%	–	34.9%	14% CKD	60% [IQR 2.5]	7%	–	0% in-hospital mortality 9.3% 1-year all-cause death
Kawsara (24)	1,513	55.7 ± 16.6	36.5%	–	–	85.9%	41%	36.2% CKD 36% liver disease	–	–	–	8.7% in-hospital mortality 26.8% cardiogenic shock

CKD, chronic kidney disease; LVEF, left ventricular ejection fraction; NYHA, New York Heart Association; RHF, right heart failure; RV, right ventricle; sPAP, systolic pulmonary artery pressure; TVR, tricuspid valve replacement.

Dreyfus et al. (28) analyzed patients treated with TV surgery in 12 French tertiary centers. Only a minority (8%, 466) underwent isolated TV surgery, and were mainly older (mean age 60 years), 24% had had previous left-sided valve surgery, ~50% presented with New York Heart Association (NYHA) class III and IV heart failure symptoms and 35% experienced heart failure within the year prior to surgery. Moreover, >50% presented with RHF, 8% with ascites and chronic kidney and liver disease were present in 33% and 12% of patients, respectively. Regarding echocardiographic data, ~20% of patients had moderate and severe RV dysfunction and a systolic pulmonary artery pressure (sPAP) ≥ 50 mmHg. More than half of patients received TV replacement. In terms of outcomes, in-hospital mortality was 10%, and at 1- and 5-years follow-up the rates of all-cause death and cardiovascular readmissions were 25 and 38%, respectively. Independent predictors associated to in-hospital mortality and mid-term follow-up were NYHA III/IV heart failure symptoms, low prothrombin time and moderate and severe RV dysfunction. These data underline the importance of timely referral. In fact, chronic severe TR leads to RV dilation and dysfunction, and when patients present with symptoms despite medical therapy it is often too late for intervention (29).

These results were further confirmed by a single-center retrospective study published by our group (26, 27). The 172 patients analyzed were divided according to a new classification based not only on TR grade, but also symptoms, RV remodeling and function, RHF episodes and medical therapy (30), ranging from Stage 1 (less than moderate TR, no symptoms) to Stage 5 (severe TR, RHF episodes despite maximal medical therapy, organ damage, severe RV dysfunction). In our experience, patients operated upon in early stages of the disease (Stage 2 and 3), without prominent symptomatology, RV dilation or dysfunction, and without organ involvement, most frequently received TV repair with no in-hospital mortality, fewer postoperative complications and shorter postoperative length-of-stay. Moreover, patients at early stages of the disease, following TR treatment, experienced 100% survival at 5 years and no further hospitalizations for RHF. On the contrary, patients in more advanced stages (Stage 4 and 5) experienced higher in-hospital mortality (15.3%), postoperative complications (such as acute kidney injury and low cardiac output syndrome), and longer both intensive care unit and hospital lengths-of-stay. In these stages, survival at 5 years was 60.5% and 20% of patients experienced at least one hospitalization for RHF following surgery.

Similar results were also reported by Weiss et al. (31) in their single-center study assessing clinical outcome and functional capacity following isolated TV surgery. Within the study, patients with severe right or left heart failure, severe pulmonary hypertension, end-stage renal disease and liver disease were excluded. No in-hospital mortality was reported and at 1-year follow-up 9% mortality was documented together with a significant improvement in functional capacity, reduction in clinically apparent peripheral edema and daily oral furosemide therapy. The population treated by Weiss et al. was highly selected and not advanced in disease progression resulting in good short-term outcomes and improved functional capacity.

On the same line are results reported by Kawsara et al. (24) that studied 1,513 patients from the Nationwide Readmissions Database, that underwent isolated TV surgery. Surrogates of late referral in the patient population were frequent, such as admission with decompensated heart failure (41%), non-elective surgery (44%), and advanced liver disease (17%). These factors were the strongest predictors of in-hospital mortality, further supporting the idea that the poor outcomes of isolated TV surgery are related to the late referral for intervention.

Even though all the recent data in the literature regarding surgical treatment of isolated TV disease stress the importance of early referral and treatment, no specific parameter and cut-off value had been identified in order to guide the decision-making process of optimal patient management. In this regard, a novel dedicated risk score has been recently made available that aims at predicting the outcome of patients following isolated TV surgery (32). The TRI-SCORE managed to identify eight parameters not only related to right and left ventricular function, but also end-organ involvement (both liver and kidney), medical therapy and clinical status. More specifically, age, NYHA functional class, RHF signs, daily dose of furosemide, renal insufficiency determined by glomerular filtration rate, elevated total bilirubin, left ventricular ejection fraction and moderate/severe RV dysfunction, were all found to be independent predictors of in-hospital mortality. Even though this scoring system still requires external validation, the TRI-SCORE, based on eight easy to ascertain parameters, is the first example of an attempt to predict favorable vs. non favorable responders to isolated TV surgery.

## Transcatheter treatment

Transcatheter treatment of severe isolated TR is becoming an accepted option for the management of patients considered high-risk or surgically ineligible. Available transcatheter treatment options mimic surgical techniques and include leaflet approximation, incomplete ring annuloplasty, heterotopic valve implantation (caval valve devices) and percutaneous tricuspid valve replacement. At present, the most widely applied technique is edge-to-edge repair of the tricuspid valve (33). Retrospective analyses have reported a reduction in TR grade, symptomatic improvement (reduced RHF hospitalizations) and lower mortality at 1 year with various devices compared to medical therapy alone (34–36). In fact, results from the TRILUMINATE trial have shown that, despite residual TR being associated with worse outcomes, reduction of at least one degree of TR is associated with improved symptoms at follow-up. Furthermore, reverse remodeling of the right ventricle, improved cardiac output and reduction of liver enzymes were also reported following TV treatment using the TriClip device (Abbott Vascular, Chicago, USA) (37–39). Results are further improving with the advent of new platforms. Despite these promising and encouraging results, it has recently emerged that, just as for surgical correction, indication and timing of any transcatheter tricuspid valve intervention (TTVI) are of paramount importance and should take into consideration multiple aspects, such as patients' clinical characteristics, disease stage, end-organ function and anatomical factors (Table 2).

**Table 2:** Characteristics of the transcatheter studies in the literature.

**Study**	**No. of patients**	**Age (years)**	**Procedure**	**NYHA III/IV**	**RHF episodes**	**End-organ involvement**	**LVEF (%)**	**≥Moderate RV dysfunction**	**sPAP (mmHg)**	**Outcomes**
Schlotter (41)	TTVI: 288 Control: 562	78 [74–82] 76 [69–82]	MitraClip, PASCAL, Trialign, Cardioband etc. None	261 (90.6%) 520 (92.5%)	–	eGFR 42 (30–58) eGFR 52 (37–71)	55 (43–61) 50 (35–60)	54% 49%	43 (34–53) 48 (37–60)	13.1% 1-year mortality 25.4% 1-year mortality
Orban (43)	75	77 [74–82]	67 MitraClip 8 PASCAL	100%	–	–	55 [49.9–62.4]	3D-RVEF 41 ± 7.8%	–	33% 1-year mortality
Brener (44)	444	76.7 ± 9.1	MitraClip, PASCAL, Trialign, Cardioband, FORMA, Tricinch, Navigate	91.4%	72.3%	eGFR 46.1 ± 20.1	50.6 ± 13.3	TAPSE 16.4 ± 4.6 mm	40.8 ± 15.3	2.3% in-hospital mortality 14.2% 1-year mortality
Lurz (49)	243	77 ± 9	MitraClip	92%	76%	eGFR 48 ± 22	51 ± 14	TAPSE 17 ± 5 mm	49 ± 15	19% 1-year morality
Stocker (50)	236	78 [74–82]	MitraClip, PASCAL	89%	–	eGFR 46 (33–59)	55 (50–60)	TAPSE 17 (13–20mm)	41 (32–49)	8% 1-year mortality with no PH; 22% 1-year mortality with post-capillary PH; 62% 1-year mortality with pre-capillary PH
Muntané-Carol (51)	300	77 ± 9	MitraClip, PASCAL, Trialign, Cardioband, FORMA, Tricinch, Navigate	93%	68.7%	eGFR 44.7 ± 20.3	49 ± 13	TAPSE 15 ± 4 mm	44 ± 17	3% in-hospital mortality 18% 6-months mortality

eGFR, estimated glomerular filtration rate; LVEF, left ventricular ejection fraction; NYHA, New York Heart Association; PH, pulmonary hypertension; RHF, right heart failure; RV, right ventricle; RVEF, right ventricular ejection fraction; sPAP, systolic pulmonary artery pressure; TAPSE, tricuspid annular plane systolic excursion; TTVI, transcatheter tricuspid valve interventions.

According to current guidelines, in patients undergoing evaluation for TR treatment, a comprehensive RV assessment should be performed, including measures of RV size and morphology, RV function and tissue remodeling (7). Non-invasive assessment of the RV is a complex task, requiring the integrated evaluation of multiple parameters, and taking advantage of emerging imaging modalities, such as speckle-tracking and 3D echocardiography or cardiac computed tomography and magnetic resonance (CMR). Nevertheless, RV dilatation and systolic function are key determinants in the evaluation and management of patients with significant TR owing to their prognostic relevance. Patients presenting with RV systolic dysfunction irrespective of RV size experience 5-years survival rates (29). Similarly, the presence of RV dysfunction has been shown to be a risk factor associated with adverse outcome in patients with TR and in tricuspid valve surgery, as underlined previously (29, 40). Schlotter et al. (41) decided to analyze the clinical impact of RV dysfunction in patients undergoing TTVI from the TriValve registry, in order to try and shed some light on favorable responders and patient selection. Patients from the TTVI cohort were compared to patients treated conservatively, and the whole population was further stratified in three subgroups according to longitudinal RV function expressed by the tricuspid annular plane systolic excursion (TAPSE): preserved (TAPSE >17 mm), mid-range (TAPSE 13–17 mm) and reduced RV function (TAPSE <13 mm). Not surprisingly, TTVI was associated with reduced mortality in patients with severe TR as compared to conservative treatment (13% vs. 25.4%, respectively). However, this survival benefit was not seen in cases of procedural failure. Even more importantly, TTVI was associated with a survival benefit solely in patients with mid-range RV function, improving their outcome to the level of patients with preserved RV function. No improvement was instead reported for patients with preserved or reduced RV function, and the latter was associated with impaired outcome in both patients treated conservatively and with TTVI. These findings may seem in contrast with those reported by Miura et al. (42), who identified RV dysfunction as an independent predictor of all-cause mortality and RHF hospitalizations in patients treated with TTVI. However, these results underline the importance of adequate timing and patient selection also in patients undergoing percutaneous procedures: patients treated in late stages of the disease, with pronounced RV dysfunction, may not benefit from the reduction in venous congestion and reverse remodeling, ultimately impacting on clinical events.

Orban et al. (43) investigated the prognostic impact of global RV function assessed using 3-dimensional (3D) echocardiography in 75 patients undergoing transcatheter tricuspid edge-to-edge repair, stratified according to preprocedural 3D RV ejection fraction (3d-RVEF). Patients in the highest tertile (3D-RVEF 44.6–61.8%) had a better survival than those in the intermediate or lower tertiles. Furthermore, at follow-up, patients in the highest RVEF tertile were more likely to be in NYHA class ≤ II and experienced greatest improvement in 6-min walking distance. Both pre-procedural RVEF and NYHA functional class IV were independent predictors of all-cause mortality. Interestingly, RV function identified by TAPSE was not predictive of outcome in these patients.

These discordant findings emphasize the complexity of non-invasive assessment of RV function by the adoption of single parameters and the need for a comprehensive evaluation. Indeed, both TAPSE and 3D-RVEF might fail to capture the actual relationship between RV contractility and afterload, leading to overestimation of RV systolic function in patients with severe TR. RV-pulmonary artery (PA) coupling helps to determine whether RV function is adequately compensated for specific loading conditions. In compensated states, RV contractile function increases together with the increase in afterload to maintain a steady RV-PA ratio. On the other hand, in decompensated states, RV contractile function does not rise together with the afterload, resulting in lower RV-PA coupling ratios. Brener et al. (44) evaluated the prognostic value of non-invasively derived RV-PA coupling in patients from the TriValve registry undergoing TTVI for severe TR. A high baseline TAPSE/systolic pulmonary artery pressure (sPAP) ratio was found to be independently associated to lower all-cause mortality with respect to lower baseline TAPSE/sPAP ratios. Furthermore, patients with higher baseline TAPSE/sPAP ratios experienced fewer hospitalizations for RHF within 12 months from TTVI treatment. Interestingly enough, the benefits associated to a high RV-PA coupling ratio were irrespective of baseline TAPSE and sPAP values, implying that this coupling measurement takes into account a contractile reserve that the single parameters are not capable of assessing.

The RV contractile reserve in response to pharmacological or physical stress has shown prognostic relevance in patients with pulmonary hypertension and severe baseline RV dysfunction, however, further studies are warranted to explore the role of stress imaging in severe TR (45).

Finally, detection of myocardial fibrosis by CMR or by speckle-tracking echocardiography has recently demonstrated prognostic importance in RV failure and might represent a promising tool to define the optimal timing of intervention in severe TR (46).

Right ventricular function and pulmonary hypertension are not the only factors responsible for an unfavorable outcome in patients undergoing TTVI. Indeed, pulmonary circulation status plays a relevant role in determining the prognosis of patients with severe TR and the outcome of TTVI. Right heart catheterization is the gold standard for the invasive assessment of the right heart, providing information regarding the severity and mechanism of pulmonary hypertension (PH), pulmonary vascular resistance, preload conditions, RV function and RV-PA coupling.

Pulmonary hypertension frequently coexists with severe TR, being a marker of poor prognosis and high operative risk (47). Furthermore, it has been shown to be responsible for adverse outcomes in patients with heart failure and patients undergoing TV surgery (48). To date, PH is often solely assessed by echocardiography. However, recent data have shown that the diagnostic sensitivity of echocardiography in accurately detecting PH is only 55%, since the determination of sPAP might be limited in severe TR (49). Lurz et al. (49) analyzed the impact of PH on clinical outcomes of 243 patients with severe TR undergoing transcatheter tricuspid edge-to-edge repair. Invasive PH (iPH) and echocardiographic PH (ePH) were defined as sPAP ≥50 mmHg. The presence of iPH resulted associated with the primary composite endpoint of death, heart failure hospitalization and re-intervention at 1 year. The echocardiographic diagnostic accuracy to detect iPH was low (55%). A discordance between non-invasive and invasive RHC assessments (iPH+/ePH-) and an impaired invasive RV-PA coupling resulted as independent predictors of the primary composite endpoint at 1 year.

The invasive cardiopulmonary hemodynamic profile predicts survival in patients undergoing TTVI, allowing risk stratification and identification of those patients that could benefit the most from intervention. Stocker et al. (50) decided to analyze RHC data of 238 patients with severe TR undergoing transcatheter tricuspid valve repair. Authors identified mean PAP, diastolic PAP, transpulmonary gradient (TPG), pulmonary vascular resistance (PVR) and right ventricular stroke work as significant hemodynamic predictors of 1-year mortality. On the other hand, pulmonary capillary wedge pressure (PCWP), right atrial pressure (RAP), cardiac output (CO), and pulmonary artery pulsatility index were not associated with 1 year mortality following TTVI. The following cutoff values were identified: mPAP >30 mmHg, sPAP >50 mmHg, dPAP >20 mmG, TPG >17 mmHg and PVR >5 WU. Moreover, stratification of patients according to mPAP and TPG resulted associated with 1 year mortality following TTVI: patients with pre-capillary dominant PH (high mPAP >30 mmHg and high TPG>17 mmHg) had an unfavorable prognosis (38% 1-year survival), while patients without or with post-capillary PH (mPAP>30 mmHg and TPG <17 mmHg) had a favorable outcome (92% and 78% survival at 1-year, respectively). These data suggest that echocardiography alone might not be sufficient in accurately detecting PH and, even more importantly, they highlight the need for a comprehensive, multimodality assessment of PH and RV function in patients undergoing TTVI. Therefore, RHC should be performed systematically as a pre-procedural assessment tool in order to better characterize TR and PH and consequently stratify patients and define their prognosis.

Recently, Muntané-Carol et al. (51) reported the outcome of a cohort of 300 patients undergoing TTVI with RV dysfunction (TAPSE <17 mm) or pulmonary hypertension (sPAP ≥50 mmHg) from the TriValve registry. Reported procedural success was 80% with 3% in-hospital mortality following TTVI. At 6 months follow-up, there was an improvement in NYHA functional class, with more than two thirds of patients in NYHA class I-II. However, at follow-up ~20% of patients died. Factors identified as independent predictors of outcome were hepatic congestion, renal dysfunction and lack of procedural success. Furthermore, the estimated 1-year mortality in patients with more advanced heart failure, with both renal dysfunction and significant hepatic congestion at baseline, was close to 50%. Therefore, transcatheter procedures may result futile in candidates with end-stage heart failure, untreated pulmonary hypertension and end-organ damage.

## The gray zones and the future

Long forgotten, the tricuspid valve has now gained great momentum. Isolated tricuspid valve treatment, both surgical and transcatheter, is matter of great debate. Even though surgery is the only definitive treatment for isolated TR, it is rarely performed in response to the historically reported high in-hospital morbidity and mortality and poor long-term outcomes (18, 52). These results have led to lengthy medical management and late referral for surgery. However, severe TR can precede right heart failure by many years until late in the natural history of the disease. This is responsible for a vicious circle that further delays or even rejects the referral for surgery. Transcatheter tricuspid treatments have therefore emerged as treatment options for severe symptomatic TR in patients considered ineligible for cardiac surgery (53). Despite numerous devices and increasing awareness of early intervention, when and in whom to perform surgical or transcatheter procedures remains a clinical conundrum. In fact, regardless the type of intervention, the ultimate focus has been on patient selection, surgical or transcatheter indication, timing of intervention and identification of predictors of outcome following treatment in order to identify favorable and non-favorable responders to treatment. The less invasive nature of transcatheter procedures, however, allow to investigate more appropriately the influence of baseline predicting factors by eliminating the influence of the surgical insult, as well as by including more patients. Transcatheter interventions will therefore help a better understanding of right heart physiology and support decision making in the future. In the future, earlier referral will also increase the rate of surgical procedures on isolated TR patients (a trend already happening in high volume centers offering transcatheter procedures).

In both treatment options, specific parameters capable of predicting outcome have been of difficult identification.

The cardiac surgery community has stressed the idea of early referral following recent data published in the literature. Early referral and early treatment, before the development of overt symptomatology, right heart dysfunction and failure and end-organ damage, are associated to excellent in-hospital outcomes, with a higher rate of TV repair vs. replacement, and good long-term outcomes, with low-to-none mortality at 5 years and significant improvement in symptomatology (24, 26, 27). To better define early referral and therefore aid in the stratification of surgical risk, the TRI-SCORE was recently developed (32). The most relevant predictors of outcome, and as a consequence of favorable and unfavorable responders, are symptomatology (NYHA class and medical therapy), end-organ involvement (hepatic congestion and renal dysfunction) and RV function [TAPSE and tissue doppler imaging (TDI)]. Interestingly enough, what has emerged in the literature, is that patient selection and optimal timing are crucial also in percutaneous tricuspid procedures. The same above-mentioned parameters were also identified as independent predictors of outcome of patients undergoing TTVI. In particular, the greatest attention in recent years was entirely directed toward the identification of the most appropriate parameter capable of defining RV function (54). Adequate assessment of RV function is extremely complex and many parameters, such as TAPSE, have given contradictory results. RV-PA coupling appears to be a powerful predictor of outcome, by assessing whether RV function is correctly compensated for specific loading conditions. Preoperative echocardiographic data concerning both right ventricular size and function are of paramount importance in order to guide when to intervene and how to treat patients with severe TR. Relevant parameters have been identified: surgery should be considered in patients with mild RV dysfunction, while transcatheter procedures result beneficial in patients with moderate RV dysfunction. However, more thorough assessment of RV function is still required, especially with the new approaches in transcatheter tricuspid valve replacement (55). In this setting, misdiagnosis of RV dysfunction may result in acute right heart failure due to sudden increase in afterload and development of afterload mismatch.

In this moment of great enthusiasm for the treatment of TR, a comprehensive evaluation by the Heart Team is mandatory, in order to thoroughly assess clinical characteristics, define the surgical and percutaneous risks and identify the most appropriate treatment strategy for each patient. However, in order to define whether either intervention is truly beneficial and in which populations, randomized controlled trials analyzing optimal medical therapy vs. surgical treatment vs. transcatheter interventions are necessary.

## Author contributions

AS made substantial contributions in the design and drafting of the work. AB made substantial contributions in revision. FM made substantial contributions by critically revising the manuscript adding important intellectual content. All authors contributed to the article and approved the submitted version.

## Conflict of interest

The authors declare that the research was conducted in the absence of any commercial or financial relationships that could be construed as a potential conflict of interest.

## Publisher's note

All claims expressed in this article are solely those of the authors and do not necessarily represent those of their affiliated organizations, or those of the publisher, the editors and the reviewers. Any product that may be evaluated in this article, or claim that may be made by its manufacturer, is not guaranteed or endorsed by the publisher.
